# Laryngeal involvement in rheumatoid arthritis

**DOI:** 10.5935/1808-8694.20130040

**Published:** 2015-11-02

**Authors:** Sarah Cristina Beirith, Claudio Marcio Yudi Ikino, Ivânio Alves Pereira

**Affiliations:** a Medical student - UFSC; b PhD in Sciences - Medical School of the University of São Paulo. Adjunct Professor - Department of Surgery - UFSC; c PhD in Medicine - Medical School of the University of São Paulo (USP). Assistant physician - Department of Rheumatology - University Hospital - Federal University of Santa Catarina (HU-UFSC). University Hospital - UFSC

**Keywords:** arthritis, rheumatoid, dysphonia, laryngeal diseases, laryngoscopy

## Abstract

**Abstract:**

The prevalence of laryngeal involvement in Rheumatoid Arthritis (RA) ranges from 13 to 75%. The specific RA manifestations include the cricoarytenoid arthritis and the presence of rheumatoid nodules in the vocal folds.

**Objective:**

The objective of this study is to evaluate the prevalence of dysphonia and laryngeal alterations on videolaryngoscopy in RA patients and their association with disease activity.

**Method:**

This is a clinical cross-sectional study that evaluated patients with rheumatoid arthritis as to their disease activity score in 28 joints (DAS-28), laryngeal symptoms, application of a Portuguese version of the Voice Handicap Index and videolaryngoscopy findings, comparing them with a control group.

**Results:**

We evaluated 47 (54%) patients with rheumatoid arthritis and 40 (46%) controls. The prevalence of dysphonia and videolaryngoscopy changes was respectively 12.8% and 72.4% in patients with RA. The mean of DAS-28 was 3.3 ± 1.2; 26 (74.3%) of 35 patients presenting active disease had laryngeal changes (*p* = 0.713). Posterior laryngitis was the most common diagnosis (44.7%).

**Conclusion:**

The prevalence of laryngeal disorders in RA patients was 72.4% and the prevalence of dysphonia was 12.8%. There was no significant relationship between laryngeal disorders and disease activity.

## INTRODUCTION

Rheumatoid arthritis (RA) is a chronic, autoimmune disease, of unknown cause, which affects mainly women between 30 and 50 years of age^1^, ^2^. The world prevalence in the adult population is approximately 0.5% to 1%, and the incidence is of 20 to 50 cases per 100,000 people annualy^1^, ^2^, ^3^. RA represents joint manifestations as well as extra-joint manifestations, which may potentially happen to any diarthrodial joint, including the cricoarythenoid^4^.

The prevalence of laryngeal repercussions in RA is 13% to 75%^5^, ^6^ in different studies and, specifically, in *post-mortem* studies, which is of 45% to 88%^6^. Knowledge on the laryngeal involvement in RA patients is longstanding: the changes were described by Mackenzie, in 1880, by means of studies in cadavers, and the first studies using laryngoscopy happened in 1960^6^.

Laryngeal manifestations in RA patients are usually subclinical^5^ and benign^6^. Initial symptoms are hoarseness, *globus* pharyngeus and the sensation of having a foreign body in the throat. Afterwards, patients experience odynophagia, sore throat, cough and dyspnea^5^. Dysphonia happens to 12% to 27% of the patients with RA, and the relative risk of dysphonia varies between 3 and 4 when compared to healthy patients^7^.

About 66% of the laryngeal symptoms in RA patients are related to cricoarytenoid joint involvement^8^. Cricoarytenoid arthritis is a potential cause of acute obstruction of the airways^9^, ^10^, ^11^, compromising vocal fold mobility and causing edema, which requires tracheostomy^12^. Other laryngeal alterations typical of RA are rheumatoid nodules, which may be mild, diagnosed only through micro-videolaryngoscopy or through histopathology, or until they become exuberant, in a bamboo shape^13^, ^14^, ^15^. Although bamboo nodules are not pathognomonic of RA, they are highly suggestive of autoimmune diseases, such as systemic erythematous lupus, Hashimoto's thyroiditis, Sjögren's syndrome and autoimmune hepatitis^14^, ^15^, ^16^. Autoimmune diseases may also cause yellow-whitish deposits, with the appearance of convex bands on the surface of the vocal folds, that is, macroscopically similar to rheumatoid nodules. Rheumatoid nodules are also part of the differential diagnosis of laryngeal cysts^16^.

Laryngeal repercussions of RA involve different specific knowledge from very distinct medical specialties: Otorhinolaryngology and Rheumatology. Notwithstanding, a large part of the papers on this topic are limited to case reports or are associated with small patient samples. Voulgari et al.^6^ reinforced the need for more prospective studies in different communities on the otorhinolaryngological and laryngeal involvements in RA. In Brazil, the knowledge regarding RA manifestations in the larynx, as well as its respective statistical data is limited. The goal of this study is to assess the prevalence of dysphonia and laryngeal changes upon videolaryngoscopy in RA patients and association with the disease's degree of activity.

## METHOD

This study was approved by the Ethics in Research with Human Beings Committee of the Federal University of Santa Catarina, under protocol number 1654. There is no disclaimer regarding this paper. This is an observational, analytical and cross-sectional study of 87 patients, 47 with RA in the study group and 40 patients in the control group. The patients were randomly invited to participate in the study and were assessed between March of 2011 and March of 2012. The individuals signed the Informed Consent Form, according to the guidelines and regulatory standards of research involving human beings.

### Series

Inclusion criteria in the study group were: the patient having a diagnosis of RA according to the American College of Rheumatology (ACR) criteria of 1987^17^. In the control group we had patients referred for videolaryngoscopy exams in the otolaryngology Ward of the University Hospital - UFSC, whom could have dysphonia and other laryngeal complaints in general, or not.

We took off the study group those patients with juvenile rheumatoid arthritis, since this form of disease has many clinical and laboratorial characteristics which are peculiar to rheumatoid arthritis in itself. In the control group we took off those patients with a confirmed diagnosis or a suspicion of RA.

### Instruments utilized

In both groups we first did an interview, which aim was to assess gender, age, proton pump inhibitors (PPI), smoking/smoking load and the presence of laryngeal symptoms in general, such as cough, dyspnea, hawking, globus pharyngeus, throat pain or irritation, vocal fatigue.

We followed by deploying the *Voice Handicap Index* (VHI) questionnaire in its translated and adapted version into Brazilian Portuguese by Jotz & Dornelles^18^. VHI is a vocal capacity self-assessment questionnaire, created with the aim of measuring the difficulties experienced by individuals with vocal disorders. The patients answered a total of 30 questions, equally divided, encompassing three aspects (functional, physical and emotional). The patients could answer the questions with never (no points), almost never (1 point), sometimes (2 points), almost always (3 points) and always (4 points), which if added up could make up a total between zero and 120 points. The cutting point on establishing dysphonia was 15 points in the total VHI scale, as well as in the study by Speyer et al.^7^.

In the RA group, we also assessed disease time and score. The *Disease Activity Score 28* (DAS-28)^19^ is a disease-activity index which assesses 28 joints in RA patients: it is obtained considering the number of painful joints and those with edema, erythrocyte sedimentation rate (ESR) and the assessment of the patient's general health. In order to establish this last parameter, the patient is asked how he/she feels regarding his/her arthritis: the patient would assign a value according to the scale in which 0 means very well and 100, very bad. According to the result obtained, we considered the patient in remittance, should the value be lower than 2.6; higher values represent some degree of disease activity^20^.

In the sequence, the patients were submitted to videolaryngoscopy, which was carried out with the 8mm 70^o^ rigid telescope, coupled to the halogen Sigmed® FL250 light source and a Toshiba® micro camera. Stroboscopy was not utilized. The 10% lidocaine spray was used in the patients' oropharynx when needed. The videolaryngoscopy exams were always done by the same examiner.

The database was built using the Epidata®, version 3.1 software, with consistency and amplitude automatic control. Following that, the data was exported to the Stata® statistical package version 11.2, in which we did the statistical analysis. The data description was expressed as absolute and relative frequencies. We then adopted the 5% (*p* < 0.05) significance value (*p*-value) for bicaudal tests. All the comparisons carried out between proportions were evaluated through the Fisher's exact test.

## RESULTS

In the group of RA patients, 40 (85.1%) were females *versus* 26 (65.0%) of the patients in the control group. Mean age was 56.5 ± 12.6 years in the RA group, and in the control group it was 47.9 ± 13.6 years. PPI was used by 35 (74.5%) of the patients in this group, and by eight (20.0%) of the patients in the control group, (*p* = 0.00). Eight (17%) patients with RA were also smokers *versus* 10 (25%) in the control group, (*p* = 0.43). The mean smoking load was 8.44 and 8.75 years/pack, respectively, between patients with RA and those in the control group.

Concerning laryngeal symptoms of patients with RA, 38 (80.9%) reported at least one symptom (Table 1) and nine (19.2%) of them denied having all the symptoms listed in the protocol. No patient in the sample reported stridor.

**Table 1:** List of the laryngeal symptoms of the patients.

Laryngeal symptoms	Rheumatoid arthritis		Control group		*p*
	n	%	n	%	
Dyspnea	08	17.02	09	22.5	0.59
Cough	22	46.81	21	52.50	0.66
Hawking	22	46.81	22	55.00	0.52
Globus pharyngeus	80	17.02	17	42.50	0.01
Throat irritation	12	25.52	14	35.00	0.35
Odynophagia	04	8.51	09	23.08	0.07
Vocal fatigue	06	12.77	14	35.00	0.02

Regarding the index suggesting the presence of dysphonia, the mean VHI among RA patients was 6.36 ± 10.82 points. In the control group, the mean was 13.15 points ± 13.78. VHI was higher than 15 points, characterizing dysphonia, in six (12.8%) of the patients with RA, and in 12 (30.0%) of the patients in the control group, (*p* = 0.064). Therefore, the ratio of dysphonia prevalence among patients in the RA group vis-à -vis the control group was 0.42.

In the RA group of patients we could see that the mean time between the disease diagnosis was 15.3 ± 8.6 years. Considering the degree of disease activity, the DAS-28 mean value was 3.34 ± 1.17. The DAS-28 was lower than 2.6 points in 12 (25.5%) patients, and higher than this cutting value in 35 (74.5%) cases.

The prevalence of videolaryngoscopy alterations in RA patients was 72.3% and, in the control group it was 65.0% (Table 2). Thus, the ratio of prevalence of such changes among patients in the RA group and that of those in the control group was 1.1.

**Table 2:** Distribution of the videolaryngoscopy results.

Videolaryngoscopy	Normal		Altered	
	n	%	n	%
RA Group	13	27.7	34	72.3
Control Group	14	35	26	65

The videolaryngoscopy diagnostics in RA patients was detailed and compared with the occurrence of patients from the control group (Table 3). One patient from the RA group had unilateral vocal fold paralysis. She reported chronic dysphonia since she was submitted to total thyroidectomy, a manifestation of a probable damage to the recurrent laryngeal nerve.

**Table 3:** List of diagnosis upon videolaryngoscopy.

Videolaryngoscopy diagnosis	Rheumatoid arthritis		Control group		*p*
Normal	n	%	n	%	0.49
	13	27.6	14	35	
Diffuse chronic laryngitis	07	14.89	10	25	0.28
Posterior laryngitis	21	44.68	13	32.5	0.27
Glottic cleft	15	31.92	01	2.5	0
Vocal nodule(s)	01	2.13	0	0	1.00
Presbylaryngitis	01	2.13	0	0	1.00
Unilateral vocal fold paralysis	01	2.13	01	2.5	1.00

We found different types of glottic clefts in RA patients: 10 (21.3%) patients had posterior triangular glottic clefts, three (6.4%) with the medium-posterior, one (2.1%) with a spindle-shaped cleft and one (2.1%) with a double cleft. One (2.5%) patient in the control group had a spindle-shape cleft.

As to the association of altered videolaryngoscopy and disease duration, 13 (27.75) patients had disease duration of less than 15 years, of whom 11 (84.6%) had normal videolaryngoscopy. Of the 34 (72.3%) patients with disease duration longer than 15 years, 18 (52.9%) had some type of change seen upon videolaryngoscopy (*p* = 0.025).

A total of 26 (74.3%) patients of the 35 with DAS-28 > 2.6 had some type of disorder seen upon the videolaryngoscopy, (*p* = 0.713). Twenty-nine (85.3%) patients with laryngeal disorders had VHI < 15 points, and five (14.7%) of the patients who had laryngeal alterations had VHI < 15 points, (*p* = 1.00).

## DISCUSSION

Both dysphonia and RA may compromise an individual's quality of life. There are just a few papers in the Brazilian medical literature discussing laryngeal changes and dysphonia in patients with RA. Since RA is a disease characterized by a course of remission and activity^1^, ^2^, it is important to correlate the videolaryngoscopy findings with the patients' clinical condition.

The RA patients in this study had moderate disease activity^19^, ^20^, with a DAS-28 score lower than what has been found in other populations. Corbacho et al.^21^, Avelar et al.^22^ and Ranzolin et al.^23^ found DAS-28 scores equal to 4.68 ± 1.79; 6.33 ± 0.92 and 4.23 ± 1.2 points. There are no studies assessing the DAS-28 relationship with the laryngeal changes. It is known that there is a direct association between the degree of disease activity and the joint and extra-joint clinical repercussion severity arising from the RA inflammation^20^.

The VHI result was lower in patients with RA (6.36 ± 10.82) vis-à -vis the control group patients (13.15 ± 13.78). The prevalence of results higher than 15 points - cutting point utilized by Speyer et al.^7^, suggesting dysphonia, was 42.3% lower in patients with RA when compared to the control group; however, this data was not statistically significant (*p* = 0.064).

Considering a recent American study assessing 55 million patients^24^, which pointed to a dysphonia prevalence in the population equal to 0.98%, one may infer that dysphonia in RA individuals is more prevalent than in the general population; however, it is less prevalent in relation to patients with laryngeal diseases and no RA. Thus, RA or its treatment may somehow impair voice production.

As to the remaining laryngeal symptoms, those patients in the control group had more vocal fatigue and globus pharyngeus than the patients with RA, and both occurrences were statistically significant (*p* = 0.02 and *p* = 0.01, respectively). Dyspnea and throat irritation were symptoms reported by patients from both groups at a similar rate (*p* = 0.59 and *p* = 0.35, respectively). Stri-dor was not reported by the patients, since it is a severe symptom - predictor of airway obstruction^9^, ^12^, which is not expected in ambulatory care level.

The prevalence of laryngeal alterations in RA patients was 72.3% and the probability of detecting changes upon videolaryngoscopy was 10% higher in RA patients when compared to those in the control group. The laryngeal alterations happened mostly to RA patients who had had the disease for over 15 years - value equal to that of the mean time of diagnosis, and the disease time higher than the mean was statistically significant in detecting laryngeal alterations in patients with RA (*p* = 0.025). Despite the cases reporting laryngeal involvement in RA patients with at least 10 years with the disease^9^, ^11^, ^18^, in the literature there is nothing saying that longer time duration of RA is a risk factor for laryngeal alterations. Results of VHI higher than or equal to 15 points were not statistically significant in relation to the laryngeal alterations seen upon videolaryngoscopy (*p* = 1.00).

The laryngeal alterations present in patients with RA did not correspond to the specific laryngeal involvement caused by the disease, represented by cricoarytenoid arthritis and by rheumatoid nodules of the vocal folds^6^, ^8^, ^13^, ^14^. None of the patients had cricoarytenoid arthritis and only one patient (2.1%) had vocal nodules. Nevertheless, we noticed that the laryngeal alterations in RA patients were similar to the ones found in patients with laryngeal diseases without RA. Thus, we may infer that RA and/or the drugs utilized to treat this disease may affect the larynx in an unspecific fashion. However, such changes did not cause so much dysphonia in RA patients as in the control group, indicating that other symptoms, such as hawking, *globus* pharyngeus and cough must suggest to the clinician a possible laryngeal involvement.

In patients with RA, posterior laryngitis was the most prevalent videolaryngoscopy-related diagnosis (44.7%), with a percentage value higher than the one found in the control group (32.5%); however, without statistical significance (*p* = 0.27). Posterior laryngitis is a common alteration caused by laryngopharyngeal reflux (LPR)^25^. In parallel to that, cough and hawking, the most prevalent symptoms in RA patients in this study, although non-specific, may also be explained by the laryngeal and pharyngeal exposure to the acid content from the stomach or some irritative factor^26^. Cough and Hawking were not significant in RA patients (*p* = 0.66 and *p* = 0.52).

PPI are important drugs in the treatment of LPR^25^, ^26^ and they are broadly utilized by RA patients (74%) (*p* = 0) because of their chronic use of non-hormonal anti-inflammatory agents^1^, ^2^, ^27^. Nevertheless, despite the use of PPI, posterior laryngitis was considered an important diagnosis in RA patients. In parallel to that, diffuse chronic laryngitis, arising from the persistence of some laryngeal mucosa irritative factor^28^, such as LPR, was found in almost 15% of the patients with RA. However, this prevalence was significantly lower when compared to the one found in the control group (25%) and there was no statistical significance (*p* = 0.28).

Although they do not represent RA-specific laryngeal alterations, glottic clefts were found mainly in these patients (31.9%), all in females, having a relevant statistical significance (*p* = 0) vis-à -vis the control group. The glottis proportion - relationship between the length of the membranous and cartilaginous portions of the vocal folds, is an important determining factor of glottic clefts: it may be reduced or enlarged - depending on gender, age and vocal abuse^29^. Presbylarynx, a type of glottic cleft in the elderly^30^, was found in a 67-year old patient with RA. Thus, since the clefts are not described as alterations pertaining to RA, they may have happened mainly in RA patients, given the female predominance^31^ in this group.

This study showed that laryngeal symptoms and laryngeal alterations are frequent in RA patients: which may indicate that RA and/or its treatment may somehow have laryngeal repercussions, even when the symptoms or morphological laryngeal involvement is not considered to be specific of RA.

## CONCLUSION

The prevalence of dysphonia, established by the VHI, and laryngeal alterations seen upon videolaryngoscopy were 12.8% and 72.3% in RA patients, respectively. The relationship between laryngeal alterations and the degree of disease activity was not established.
